# Oxidative Stress in Brain Function

**DOI:** 10.3390/antiox14030297

**Published:** 2025-02-28

**Authors:** Daniela-Marilena Trofin, Dragos-Petrica Sardaru, Dan Trofin, Ilie Onu, Andrei Tutu, Ana Onu, Cristiana Onită, Anca Irina Galaction, Daniela Viorelia Matei

**Affiliations:** 1 Doctoral School, University of Medicine and Pharmacy “Grigore T. Popa”, 700454 Iasi, Romania; 2 Department of Biomedical Sciences, Faculty of Medical Bioengineering, University of Medicine and Pharmacy “Grigore T. Popa”, 700454 Iasi, Romaniailie.onu@umfiasi.ro (I.O.); daniela.matei@umfiasi.ro (D.V.M.)

**Keywords:** oxidative stress, Parkinson disease, brain function, Alzheimer disease, multiple sclerosis, stroke, physical exercise

## Abstract

Oxidative stress (OS) is an important factor in the pathophysiology of numerous neurodegenerative disorders, such as Parkinson’s disease, multiple sclerosis, cerebrovascular pathology or Alzheimer’s disease. OS also significantly influences progression among the various neurodegenerative disorders. The imbalance between the formation of reactive oxygen species (ROS) and the body’s capacity to neutralize these toxic byproducts renders the brain susceptible to oxidative injury. Increased amounts of ROS can result in cellular malfunction, apoptosis and neurodegeneration. They also represent a substantial factor in mitochondrial dysfunction, a defining characteristic of neurodegenerative disorders. Comprehending the fundamental mechanisms of OS and its interactions with mitochondrial function, neuroinflammation and cellular protective pathways becomes essential for formulating targeted therapeutics to maintain brain health and reduce the impacts of neurodegeneration. We address recent highlights on the role of OS in brain function in terms of significance for neuronal health and the progression of neurodegenerative disorders.

## 1. Introduction

The brain is particularly vulnerable to oxidative stress (OS) because of its high energy requirements and metals such as iron and copper, which, combined with hydrogen peroxide, generate the hydroxyl radical. The hydroxyl radical is one of the most reactive and damaging free radicals, capable of severely damaging essential cellular components such as proteins, lipids and DNA [1,2,3].

Under conditions of chronic OS, the balance between the production and neutralization of reactive oxygen species (ROS) is disrupted, allowing them to accumulate in cells and tissues. Neurons are vulnerable to OS because of high oxygen consumption and limited antioxidant resources and are susceptible to damage and dysfunction, contributing to the onset and progression of neurodegenerative diseases. ROS or redox-active metal overload is regulated by cellular defense mechanisms and natural antioxidants, which have important physiological roles in the brain [4,5,6,7].

Mitochondrial dysfunction is among the primary causes of OS in the brain. Neurons are especially susceptible to oxidative aggression because of their increased metabolic activity and dependence on aerobic respiration, which produces ROS as byproducts. Elevated ROS generation can result in lipid peroxidation, protein oxidation and DNA damage, all of which contribute to neuronal injury and mortality [8].

Moreover, the brain’s distinctive lipid composition, marked by elevated concentrations of polyunsaturated fatty acids, renders it especially vulnerable to oxidative damage. Lipid peroxidation is a significant process that transpires when ROS targets fatty acids, resulting in the production of reactive aldehydes that can intensify OS and brain injury [8]. The buildup of oxidized lipids and proteins can establish an undesirable cycle, sustaining OS and facilitating the development of neurodegenerative disorders, including Alzheimer’s disease (AD) and Parkinson’s disease (PD) [9].

Inflammation is a major contributor to OS in the brain, as neuroinflammatory responses can activate glial cells that generate pro-inflammatory cytokines and ROS [10]. This inflammatory state can harm neuronal cells and compromise the blood–brain barrier (BBB) [10].

Additionally, extrinsic variables, including environmental pollutants, stress and trauma, may potentially exacerbate OS in the brain. Traumatic brain injury is also linked to elevated ROS generation and ensuing oxidative damage [11]. Psychological stress is associated with increased OS indicators in the brain, indicating that both acute and chronic stressors may intensify oxidative processes [12,13].

The origins of OS in the brain are multifaceted, encompassing mitochondrial dysfunction, lipid peroxidation, neuroinflammation and external stressors, all of which perpetuate a harmful cycle of oxidative damage, a central pathophysiological aspect within the various neurological disorders to be further discussed.

Understanding the mechanisms of the brain’s adaptability to OS is essential in establishing treatment protocols for various pathologies, such as PD and dementia (especially AD), and demyelinating pathologies, such as multiple sclerosis (MS). Cerebrovascular pathology and post-stroke rehabilitation also represent important physiopathological aspects.

Alongside conventional antioxidants, innovative methods employing nanotechnology and targeted delivery systems are being investigated to improve the effectiveness of antioxidant treatments. Single-atom nanozymes have been engineered to adsorb ROS and reactive nitrogen species (RNS), offering a novel approach to decrease OS in the brain [14].

Although the potential of antioxidants in addressing neurological disorders is encouraging, obstacles persist in applying these discoveries in therapeutic settings. The variability in individual responses to antioxidant therapies and the complexity of OS pathways in diverse neurological conditions require additional research to optimize treatment regimens and identify patient populations that may derive the greatest benefit from antioxidant interventions [15,16].

## 2. Pathophysiological Mechanisms by Which the Brain Adapts to Oxidative Stress

The brain’s response to OS is a complex process involving several pathways. OS, defined as an imbalance between the generation of ROS and antioxidant defenses, significantly contributes to neurodegenerative disorders and cognitive loss. The mechanisms by which the central nervous system (CNS) manages to adapt involve certain pathways and the use of various specific proteins, which elicit interest and ongoing research as we speak.

Glutamate, a major excitatory neurotransmitter, is involved in synaptic plasticity and excitotoxic cell death. It binds to receptors like the N-methyl-d-Aspartate receptor (NMDAR), a calcium channel in synaptic and extrasynaptic sites. Calcium entry through extrasynaptic NMDARs leads to calcium overload in mitochondria, which contributes to neuronal death. Disruption of calcium homeostasis triggers neuronal death, contributing to diseases like AD and PD [17].

The brain primarily adjusts to OS by activating neuroprotective signaling pathways. The nuclear factor erythroid 2-related factor 2 (Nrf2) pathway is essential for cellular protection against oxidative effects. The activation of Nrf2 induces the transcription of multiple antioxidant genes, hence augmenting the brain’s capacity to combat OS [18]. Notoginsenoside R1 can activate the Nrf2 pathway, offering neuroprotection against neuronal injury. Likewise, paraoxonase 2 (PON2) has been associated with neuroprotection by regulating OS responses via Nrf2 activation [18,19]. The interplay between DJ-1 (a protein linked to PD) and PON2 highlights the significance of both pathways in neuronal survival [20].

The phosphoinositide 3-kinase (PI3K)/Akt signaling pathway, alongside the Nrf2 pathway, serves as a crucial mechanism for neuroprotection. This pathway is implicated in cell survival and anti-apoptotic signaling. Thioflavones activate the PI3K/Akt pathway, resulting in apoptosis suppression and increased cell survival under OS conditions [21]. The activation of this pathway is influenced by sex hormones, including testosterone and estrogen, which have demonstrated protective effects in neuronal cells [22]. Interaction among these signaling pathways is what actually underscores the brain’s adaptive responses.

The involvement of particular proteins in facilitating neuroprotection is noteworthy. DJ-1 is recognized for its oxidation under stress conditions, which is crucial for its neuroprotective functions [23]. Oxidation of DJ-1 increases its interaction with PON2, thereby facilitating neuronal survival under OS conditions [20]. The expression levels of PON2 are influenced by gender, with higher levels found in females, indicating that hormonal differences may impact the brain’s vulnerability [24]. This gender-specific response suggests that the brain’s adaptation mechanisms are influenced by both biochemical factors and biological sex.

The involvement of neuroprotective compounds is another critical aspect of the brain’s adaptation to aggression. Natural compounds like quercetin and astaxanthin exhibit antioxidant properties that may reduce oxidative damage in the brain. Quercetin enhances the brain’s capacity to alleviate OS-induced toxicity through the modulation of PON2 levels [25]. Astaxanthin has been shown to decrease brain aging through the reduction in OS and the enhancement of brain-derived neurotrophic factor (BDNF) levels, essential for neuronal health and function [26]. Such findings indicate that dietary and pharmacological interventions could improve the brain’s resilience to OS; therefore, we discuss them in dedicated subtopics.

The neuroprotective effects of erythropoietin (Epo) and its variants, including EpoL, have attracted interest due to their capacity to activate anti-apoptotic pathways and decrease ROS levels in neuronal cells [27,28]. The mechanism of action of Epo entails the activation of the erythropoietin receptor (EpoR), which results in the phosphorylation of signaling molecules that enhance cell survival. This receptor-mediated neuroprotection underscores the potential of therapeutic agents that target these pathways to improve the brain’s adaptive responses. The presence of endocannabinoids influences the brain’s response to OS, demonstrating protective effects against oxidative damage. Endocannabinoids can influence responses in neural progenitor cells, indicating a protective function in preserving neuronal health during stress conditions [29].

The neuroprotective effect may be facilitated by the activation of many signaling pathways that enhance cell survival and diminish apoptosis. Furthermore, the brain’s resistance is associated with its metabolic condition. The metabolism of dopamine in particular brain areas, including the striatum, correlates with elevated OS resulting from the generation of ROS during dopamine metabolism [24]. This inherent sensitivity requires strong antioxidant defenses, typically facilitated by proteins such as PON2, which are increased in response [19]. Comprehending the regional variations in responses can yield further insights into specialized therapeutic approaches for neuroprotection.

The initiation of inflammatory pathways results in neuronal damage and aggravation of neurodegenerative mechanisms, like in the case of activation of NF-κB, a transcription factor associated with inflammatory reactions that have been correlated with OS in the nervous system [30]. NF-κB demonstrates neuroprotective qualities, indicating a dual role in the brain’s response [30]. This complexity highlights the necessity for a sophisticated comprehension of the interplay of OS, inflammation and neuroprotection. This is the context in which the significance of dietary antioxidants comes into play in promoting the brain’s resistance. Compounds originating from natural sources, such as sterols from Mytilidae, demonstrate antioxidative characteristics that enhance neuroprotection [31]. Findings like this indicate that dietary changes may significantly influence OS responses in the brain, potentially providing preventive methods against neurodegenerative disorders.

**Figure 1:**
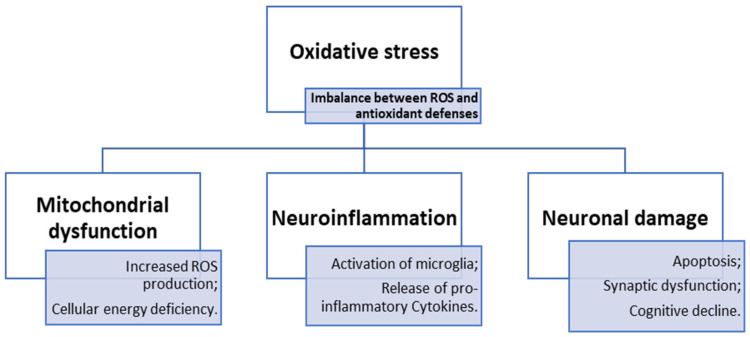
Schematization of the role of oxidative stress in neurodegenerative diseases.

Briefly, the main mechanisms that lead to the cascade of events that cause multiple pathologies in the brain can by synthetized in Figure 1. Going into detail, the brain’s response to OS is based on a complex interaction of signaling pathways, particular proteins and neuroprotective agents. Nevertheless, not only the activation of pathways such as Nrf2 and PI3K/Akt, or the involvement of proteins like DJ-1 and PON2, modulate the brain’s response to OS, but also dietary antioxidants and neuroprotective properties of different studied compounds, which rise more and more interest in terms of prevention and treatment of neurodegenerative disorders.

## 3. Oxidative Stress in Parkinson’s Disease

The degeneration of the substantia nigra pars compacta is the pathological hallmark of PD, with specific motor symptoms manifesting after around 60% of the neurons that produce dopamine are lost [32]. When dopaminergic neuron cell loss occurs inside the substantia nigra, the quantity of particular dopamine nerve terminals in the striatum diminishes, and movement execution is altered in terms of rigidity and akinesia. The depletion of dopamine induces a diminished activity in the direct pathway and an enhanced activity in the indirect pathway of the cortico-striato-pallido-thalamo-cortical circuit [32,33]. The disinhibition of primary output nuclei and heightened inhibition of the thalamocortical system lead to the characteristic pill-rolling tremor [32].

The reduction in dopaminergic neurons in PD diminish the activity at the globus pallidus internus (Gpi) and substantia nigra pars reticulata (SNr), resulting in heightened inhibitory output from both Gpi and SNr. The dopamine deficit disinhibits striatopallidal neurons synapsing in the globus pallidus externus (Gpe), hence diminishing the activity of the inhibitory pallidosubthalamic neurons. Dopamine depletion enhances striatal activity through projections to GABAergic neurons, which amplify actions on the Gpe. Moreover, the depletion of dopamine also results in disinhibition of the subthalamic nucleus [32,33,34].

ROS contributes to neuronal degeneration in PD. OS significantly contributes to the degradation of dopaminergic neurons in the substantia nigra. The enzymatic oxidation of dopamine by monoamine oxidase-B results in the production of hydroxyl free radicals, especially in the presence of iron, which is prevalent in the basal ganglia [35,36]. This mechanism leads to dopamine depletion and the production of toxic metabolites that worsen neuronal damage. The susceptibility of dopaminergic neurons to OS is exacerbated by their inherent metabolic characteristics, such as the existence of ROS-generating enzymes like tyrosine hydroxylase. This vulnerability indicates that OS is a pivotal element in the etiology of PD, resulting in neuronal cell death and the hallmark motor symptoms linked to the condition.

There are particular signaling pathways that regulate the responses in PD. The Nrf2 pathway is one of the most significant because it governs the development of antioxidant proteins that safeguard against oxidative damage [37]. The activation of Nrf2 has demonstrated the ability to rectify metabolic deficits in patient-derived cells exhibiting sporadic PD, suggesting its viability as a therapeutic target [37]. Moreover, the deregulation of Nrf2 in PD patients is associated with elevated OS levels, indicating that the enhancement of this pathway may confer neuroprotective benefits [37,38].

Besides Nrf2, the significance of sirtuin 1 (SIRT1) has been emphasized regarding OS in PD. SIRT1 is recognized for reducing neuronal cell death and is downregulated in PD [39]. Modulating SIRT1 activity may serve as a viable approach to minimize the harmful oxidative consequences on dopaminergic neurons. Furthermore, the relationship with neuroinflammation has been confirmed, with persistent inflammation exacerbating neuronal loss in PD [40,41].

The activation of microglia and the secretion of pro-inflammatory cytokines can intensify OS, establishing a detrimental cycle that speeds up neurodegeneration [42]. Mitochondrial failure in PD is intricately associated with these processes. Mitochondria serve as both a generator and a target of ROS, as their malfunction may result in heightened oxidative damage in neurons [43].

Mitochondrial complex I impairment appears to be common in PD, leading to the accumulation of ROS and consequent neuronal death [44]. The correlation between mitochondrial malfunction and OS highlights the significance of focusing on mitochondrial health as a prospective therapeutic strategy in PD. Environmental factors, including exposure to pesticides such as paraquat and rotenone, are already known to be associated with parkinsonism via pathways related to OS [45,46]. These chemicals elicit effects by impairing mitochondrial activity and enhancing the production of ROS, resulting in the degeneration of dopaminergic neurons. Epidemiological studies indicate an association between pesticide exposure and an elevated chance of developing PD, underscoring the necessity for additional exploration of environmental factors contributing to oxidative stress in this scenario [45].

N-acetylcysteine (NAC) is also a recognized antioxidant that restores glutathione levels and exhibits neuroprotective properties in multiple models of brain damage and neurodegeneration [47]. In a study conducted by Pandya et al., NAC demonstrated enhancement in bioenergetics and behavioral outcomes post-traumatic brain damage (TBI), underscoring its potential as a therapeutic agent [47]. Likewise, resveratrol, an additional antioxidant, has been documented to safeguard dopaminergic neurons against oxidative stress-induced apoptosis in PD mice [48]. These findings highlight the therapeutic potential of pharmaceutical antioxidants in the management of oxidative stress-related neurological disorders.

Dietary polyphenols, including curcumin, have also been studied for their capacity to diminish OS and enhance neuronal survival in PD models [49]. Such compounds may augment the brain’s antioxidant defenses and offer a supplementary strategy to conventional pharmaceutical therapies. Identifying biomarkers for OS in PD is essential for early diagnosis and tracking disease progression. The reduction in cysteinyl-glycine levels has been suggested as an indirect biomarker for OS in parkinsonian patients receiving levodopa medication [50]. These biomarkers could enable prompt treatments and enhance patient outcomes by permitting the evaluation of OS levels in clinical environments.

## 4. Oxidative Stress in Multiple Sclerosis

The inflammatory aspect of MS affects the brain and spinal cord, with numerous genetic correlations currently under investigation, alongside efforts to slow down disease progression, particularly through relapse prevention. Neurodegeneration and symptomatology in MS are attributable to ion channel dysfunctions [51]. Pathological Na+ channel blockade occurs during acute inflammatory episodes, alongside the role of heightened Na+ channel expression in the neurodegenerative cascade. The demyelination process is also based on the physiopathology of K (+) channels and the control of rapid K (+) conductance; in other words, alterations in axonal excitability that revert to normal levels result in symptom amelioration [52].

Pathophysiological mechanisms in MS associated with vitamin D deficiency suggest an association between vitamin D sources, including dietary intake and sunlight exposure, and the incidence of MS. Considering the environmental factors associated with low vitamin D levels, various studies on animal models of demyelinating pathology related to vitamin D, along with immune and genetic perspectives, it is increasingly evident that vitamin D is assuming a significant role in the management of MS by the involvement of OS and inflammation [53,54]. Vitamin D insufficiency has been correlated with elevated levels of pro-inflammatory cytokines and OS indicators in persons with MS [55]. This indicates that inadequate vitamin D may intensify oxidative damage in the CNS, resulting in clinical symptoms and disease development. Moreover, vitamin D is believed to augment the expression of antioxidant enzymes, thereby diminishing oxidative stress and its related harm [56].

Vitamin D administration can reduce OS indicators and enhance antioxidant status in experimental models of MS [57]. The capacity of Vitamin D to regulate calcium homeostasis and affect mitochondrial activity may also play a role in its protective effects against OS [57]. The activation of the vitamin D receptor (VDR) is essential for mediating these effects, as VDR activation has been demonstrated to augment the expression of genes associated with antioxidant defense systems [58].

For all that, OS implication in MS is complex, involving the activation of immune cells like macrophages and microglia that produce ROS during inflammation. These ROS contribute to mitochondrial malfunction, neuroinflammation and ultimately, neuronal injury [59,60]. Oxidative damage is common, especially in regions of early demyelination, where apoptotic oligodendrocytes are often detected. This oxidative damage compromises myelin integrity and fosters a pro-inflammatory setting, aggravating the illness process [61,62]. Increased amounts of ROS along the multiple signaling pathways can activate the mitogen-activated protein kinases (MAPKs), amplifying inflammatory reactions [59,60].

This vicious cycle of OS and inflammation contributes to the chronic nature of MS, as inflammatory mediators perpetuate oxidative damage, leading to a decline in neuronal function and survival [63,64]. The presence of lipid peroxidation products, such as malondialdehyde, has also been investigated, indicating that lipid metabolism may play a crucial role in the disease’s progression [60,62]. Furthermore, the brain is highly dependent on mitochondrial function for energy production, the pathophysiological context in which OS can lead to mitochondrial injury, resulting in energy failure within the CNS [64,65]. This energy deficit is particularly detrimental to oligodendrocytes, which are essential for myelin maintenance and repair. The liberation of iron from damaged myelin during demyelination further exacerbates the negative impact, as free iron can catalyze the formation of highly reactive hydroxyl radicals through Fenton chemistry [65,66]. The resulting oxidative damage not only affects myelin but also contributes to neuronal degeneration, highlighting the implications and inter-relations between OS, mitochondrial dysfunction and neuroinflammation in the demyelinating process.

The activation of the Nrf2 pathway, which governs the expression of antioxidant proteins, has been suggested as a method to bolster the brain’s antioxidant defenses [66]. The application of drugs that stimulate Nrf2 activation may provide neuroprotective benefits by diminishing oxidative damage and inflammation in MS [66].

Also, dietary antioxidants and pharmaceutical medicines that alleviate OS may function as supplementary therapies to standard treatments, thereby enhancing patient outcomes [67,68].

Identifying biomarkers associated with OS in MS is essential for comprehending disease progression and treatment effectiveness. Assessing OS markers, including glutathione and malondialdehyde, may yield insights into the inflammatory condition of MS patients [63,69]. The correlation between OS and impairment in MS patients highlights the necessity of tracking oxidative damage as a possible measure of disease severity and treatment efficacy [62,69].

Elevated serum levels of vitamin D are linked to a diminished chance of acquiring MS and may also correspond with enhanced clinical outcomes in affected people [70,71]. Although substantial evidence connects vitamin D insufficiency to OS and MS, so far, there are also some inconclusive results concerning the robustness of this correlation. Wang et al. discovered very minor correlations between vitamin D deficiency and OS in specific groups, indicating that additional factors, such as age and pre-existing conditions, may affect these linkages [72]. Vitamin D supplementation may modulate OS and provide therapeutic advantages for persons with MS; however, additional research is still required to determine the appropriate dose and treatment regimens. Comprehending the pathways that connect vitamin D and OS in MS becomes relevant for formulating targeted therapies to enhance patient outcomes.

## 5. Oxidative Stress in Stroke

OS also plays an important role in the pathophysiology of ischemic stroke by leading to cellular damage, significantly contributing to neuronal injury pre- and post-ischemic events, especially related to the disruption of cerebral blood flow. This leads to hypoxia and subsequent reperfusion injury. Along with this, there is an increased generation of free radicals [73]. This is the way through which the deficiency of oxygen in brain cells elevates the risk of cerebrovascular events, especially by exacerbating inflammatory phenomena [74].

The vascular architecture, particularly by the presence of the circle of Willis, can influence the distribution of blood flow and the extent of ischemic damage, as variations in cerebral vascular anatomy can affect the risk and outcomes of stroke, all these suggesting that certain anatomical configurations may predispose individuals to more severe OS during cerebrovascular events [75,76]. Furthermore, the brain’s unique susceptibility to oxidative damage is partly due to its high metabolic rate and oxygen consumption: the induction of NADPH oxidase, particularly NOX4, is significantly elevated following ischemic events, contributing to OS and neurodegeneration [77].

OS levels in stroke patients can be assessed with biomarkers, with malondialdehyde (MDA) being one of the most frequently cited. MDA is a byproduct of lipid peroxidation and serves as a reliable indicator of oxidative aggression, as elevated MDA levels correlate with reduced muscle strength and physical performance in post-stroke patients [74]. Also, excessive OS contributes to the formation of unstable atherosclerotic plaques, significant precursors to ischemic stroke [78]. This is, therefore, a significant mediator of neuronal loss in cerebral ischemia, contributing to the propagation of injury [79]. This oxidative damage is particularly pronounced in the penumbra region, where the balance between cell survival and death is precarious. These findings underscore the multifaceted role of OS in both the acute and chronic phases of stroke, especially as Thioredoxin, a blood biomarker for assessing oxidative damage in acute ischemic stroke, can be measured for diagnostic and prognostic capabilities [80].

These parameters are also significantly elevated in hemorrhagic stroke patients, reinforcing the notion that OS is a common pathway in cerebrovascular events [81]. The extravasation of blood into the brain tissue triggers a cascade of inflammatory responses and the production of ROS throughout changes that lead to BBB disruption, edema and neuronal cell dysfunction, exacerbating the initial injury [82]. The imbalance between ROS production and antioxidant defenses contributes to vascular injury, particularly in the context of hypertension [83].

Oxidative aggression peaks within the first few days post-stroke, with significant increases in biomarkers such as F2-isoprostanes, which are indicative of lipid peroxidation [84]. This temporal aspect is crucial for understanding the window of opportunity for therapeutic interventions aimed at lowering oxidative effects. Furthermore, a correlation between increased OS levels and elevated prolidase enzyme activity in patients with acute hemorrhagic stroke suggests oxidative influence upon enzymatic pathways involved in tissue repair and remodeling [85].

The role of antioxidants in the management of OS in hemorrhagic stroke is gaining attention, especially green tea extracts, rich in polyphenols, which are believed to exhibit neuroprotective effects by reducing oxidative activity and inflammation in models of hemorrhagic stroke [86]. This, along, of course, with the potential of dietary antioxidants in modulating oxidative impact and improving outcomes in stroke patients [87]. Dietary interventions or supplementation with antioxidants may be a viable strategy to enhance recovery and reduce secondary brain injury in hemorrhagic stroke patients.

The interplay between OS and neuroinflammation following intracerebral hemorrhage (ICH) contributes to neuronal apoptosis and necrosis [88]. This relationship underscores the need for therapeutic strategies that target both oxidative damage and inflammatory pathways to improve clinical outcomes. The potential of heme oxygenase-1 (HO-1) as a biomarker and therapeutic target is also to be considered, as elevated serum levels of HO-1 have been associated with better outcomes in patients with intracerebral hemorrhage [89].

Coming back to ischemia as an etiology of stroke, the role of genetic variations in OS-related genes and their influence on ischemic stroke susceptibility is also a notion of recent interest as the individual responses to oxidative activity may vary, potentially informing personalized treatment strategies [90]. On the other hand, the interplay between OS and mitochondrial dysfunction has been documented, as oxidative damage to mitochondrial DNA can lead to impaired cellular function and also contribute to the pathogenesis of ischemic stroke [91]. Therapeutic strategies targeting OS are gaining traction as potential interventions for stroke management by including the use of antioxidants and other pharmacological agents [92].

The potential of polyphenols as antioxidant supplements has also been explored in cerebrovascular pathology, with evidence suggesting that they may provide neuroprotection by counteracting oxidative reactions [93]. This therapeutic avenue is particularly relevant given the growing body of evidence linking OS to adverse functional outcomes post-stroke rehabilitation [94].

OS not only damages cellular structures but also promotes inflammatory responses, which can further aggravate cerebral injury following ischemic stroke. This dual role of OS necessitates a multifaceted approach to treatment, addressing both oxidative damage and inflammatory pathways [95].

Considered an important risk factor for many neurological pathologies, including stroke and related dementia syndromes, alcohol-induced toxicity may worse the degradation. Lately, mitochondrial dysfunction in ethanol neurotoxicity research has opened new pathways for understanding neuron dysfunction mechanisms and potential pharmacological targets to reduce ethanol-induced neuronal loss, impacting synaptic transmission, memory and learning, affecting neuron damage and death [96].

Another risk factor (linked to alcohol consumption, either chronic or binge-drinking) for brain diseases (both acute or exacerbation of chronic ones) is trauma. Traumatic brain injury can cause damage that leads to the premature development of underlying chronic disorders. Carvajal, Mattinson and Cerpa assert that the distribution of NMDAR is important in the exacerbation of chronic pathologies based on modification in the distribution of NMDARs (AD, PD, and even Huntington’s disease and amyotrophic lateral sclerosis). Extrasynaptic NMDARs are activated by glutamate spillover, resulting in calcium overload linked to excitotoxicity, neuronal injury and mortality. The signaling involved in intermediary processes is modified, particularly the calcium-mediated signaling and the interactions with various pathways, therefore offering a range of potential therapeutic targets to regulate the neurotoxicity associated with NMDARs [97].

## 6. Dementia and Oxidative Stress Modulation by Medication and Natural Antioxidants

**Figure 2:**
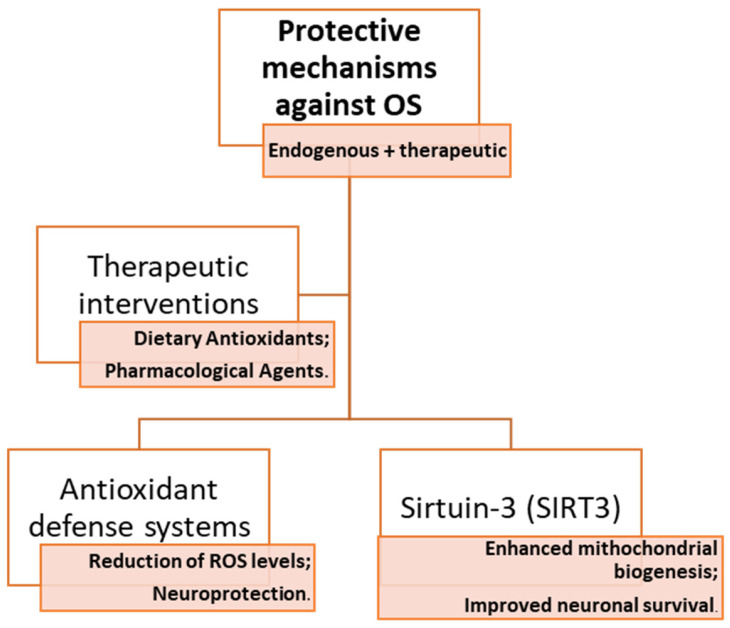
Protective mechanisms of the brain against oxidative stress effects.

Just like PD, MS or cerebrovascular diseases, by the pathophysiological mechanisms activated by OS, dementia has been intensively studied for applicability of therapeutic strategies, as well as pharmacological solutions and dietary perspectives (algorithm summarized in Figure 2).

The mitochondrial deacetylase known as Sirtuin-3 (SIRT3) is believed to play a crucial part in the mechanisms of OS that are related to dementia. There is evidence that mitochondrial damage is present in AD patients since its expression is altered [98]. Based on the findings of Weir et al., SIRT3 appears to play a crucial role in the regulation of ROS levels within neurons [99]. The increase in SIRT3 in response to amyloid-beta (Aβ) toxicity suggests the existence of a compensatory mechanism that can offset the effects of OS. SIRT3 is also responsible for mediating the deacetylation of SOD2, which in turn boosts the activity of SOD2 and encourages the scavenging of ROS [100,101]. For neuronal protection against oxidative damage, which is common in dementia-related diseases, this function is very needed. Furthermore, SIRT3 has been shown to play a role in the regulation of tau protein levels [98,99]. The protective benefits of SIRT3 are also derived from the increase in mitochondrial integrity and function [98]. Ren et al. found a correlation between the decrease in SIRT3 function and the apoptosis and degeneration of specific neuronal populations [102]. Despite all of this, SIRT3 plays an important role as a mediator in maintaining the equilibrium between neuroprotection and oxidative stress, which highlights its potential as a therapeutic target in dementia.

Development of medication in neurodegenerative diseases such as PD and MS, along with dementia (especially AD), where oxidative damage targets lipids, proteins and DNA, has yielded much interest throughout the last decades [14,15,36].

The main pharmacological medications employed in the treatment of AD consist of cholinesterase inhibitors and NMDA receptor antagonists. Cholinesterase inhibitors, including donepezil, rivastigmine and galantamine, are formulated to augment cholinergic neurotransmission by blocking the enzyme acetylcholinesterase, which is responsible for the degradation of acetylcholine in the synaptic cleft [103]. The increase in acetylcholine levels correlates with enhancements in cognitive performance and daily living activities in individuals with mild to moderate Alzheimer’s disease, with good tolerability of the three agents among patients [103,104]. These drugs can elicit enhancements in cognitive and functional results, although the benefits may differ among individuals. Association with vitamin E may also enhance synergism with better effects [103].

Choline, phosphatidylserine, citicoline and choline alphoscerates contribute to memory consolidation. Choline is a crucial component in the formation of phospholipids and serves as a precursor for acetylcholine. Decreased phosphatidylcholine levels in neurons may also contribute to cognitive deterioration [105]. Citicoline is a chemical essential for the creation of phosphatidylcholine and may be depleted when the body requires increased production of acetylcholine. The synthesis of phosphatidylcholine seems to be disrupted by ischemic stroke, resulting in individuals being at double the risk of developing dementia [106]. Supplementation of citicoline at a dosage of 500 mg daily for a duration of 9 months is advantageous for individuals with mild vascular cognitive impairment [106].

Alongside cholinesterase inhibitors, the primary glutamatergic approach for AD is the non-competitive NMDA antagonist memantine, which exhibits a modest affinity for the NMDA receptor and is characterized by a quick onset and offset of activity, which is often associated with moderate to severe AD [107]. Mira and Cerpa assert that while calcium influx via NMDA receptors, most likely primarily through extrasynaptic NMDA receptors, serves as the principal source of calcium-inducing mitochondrial dysfunction in cultured neurons, this has not been evidenced in pathological tissues, indicating that both mechanisms are closely interconnected, nevertheless assessable through indirect methodologies [17,108].

Glutamate is the primary excitatory neurotransmitter associated with learning, memory and cognition. Suboptimal concentrations of Aβ oligomers facilitate the activation of specific glutamate receptors, notably mGlu5 and NMDA receptors carrying the GluN2B component [109]. This activation may elevate intracellular Ca^2+^ levels, hence facilitating excitotoxicity and neuronal apoptosis. Glutamatergic stimulation may also facilitate the suppression of long-term potentiation (LTP) of AMPA receptors [109]. A decrease in the quantity of AMPA receptors diminishes rapid excitatory transmission and may lead to synaptic loss [109,110].

Memantine modulates glutamate activity, improving learning and memory mechanisms. Memantine might reduce neuronal damage and enhance cognitive function by inhibiting excessive glutamate activation [104,107]. Besides cognitive performance, it helps overall functioning, especially in patients currently undergoing treatment with cholinesterase inhibitors [103,107].

The combination of cholinesterase inhibitors and memantine can provide enhanced advantages when compared to monotherapy. Synergic donepezil and memantine showed superior enhancements in cognitive performance and daily life activities compared to patients receiving either medication individually, advocating that comprehensive therapy strategies may be advantageous in addressing the intricate symptoms of AD [103,111].

Regarding nicotinic agonists, the activation of α7-nAChR may facilitate the precise control of cholinergic transmission in the brain, potentially resulting in cognitive enhancement and the amelioration of AD [112].

New therapeutic targets and molecules designed to alter the disease’s trajectory are also currently being investigated. Amyloid-beta-targeting treatments, including monoclonal antibodies, are being researched for their ability to diminish amyloid plaque deposition in the brain, a characteristic feature of AD pathogenesis.

Among dietary natural antioxidants that have been investigated for their neuroprotective effects, polyphenols present in fruits like Mangifera indica (mango) have demonstrated the ability to enhance cognitive function and diminish OS in animal models of moderate cognitive impairment [113]. Phenolic chemicals in mango fruit extracts are associated with improved memory performance and decreased oxidative damage, indicating that dietary interventions may contribute to neuroprotection [114].

Moreover, walnuts (Juglans regia) have been recognized as a source of natural antioxidants that may possess neuroprotective properties, evidenced by their capacity to regulate oxidative stress and inflammation [115].

The effectiveness of antioxidants in clinical environments is additionally corroborated by research examining their impact on particular neurological disorders. Vitamin E, a recognized antioxidant, has been linked to enhanced cognitive performance in aging individuals and patients with AD [116]. Its preventive function against oxidative influence is especially pertinent considering the heightened oxidative load noted in neurodegenerative disorders. Furthermore, the activation of the Nrf2 pathway has been suggested as a therapeutic approach to augment the brain’s antioxidant defenses, with substances such as sulforaphane demonstrating potential in preclinical models of PD [117].

Lifestyle modifications such as cognitive training, physical exercise or social interaction may enhance cognitive functioning and quality of life for patients with dementia, especially AD, as combining non-pharmacological techniques with pharmacological treatments may offer a more comprehensive disease-management strategy.

## 7. Physical Exercise and Oxidative Stress in the Brain

**Figure 3:**
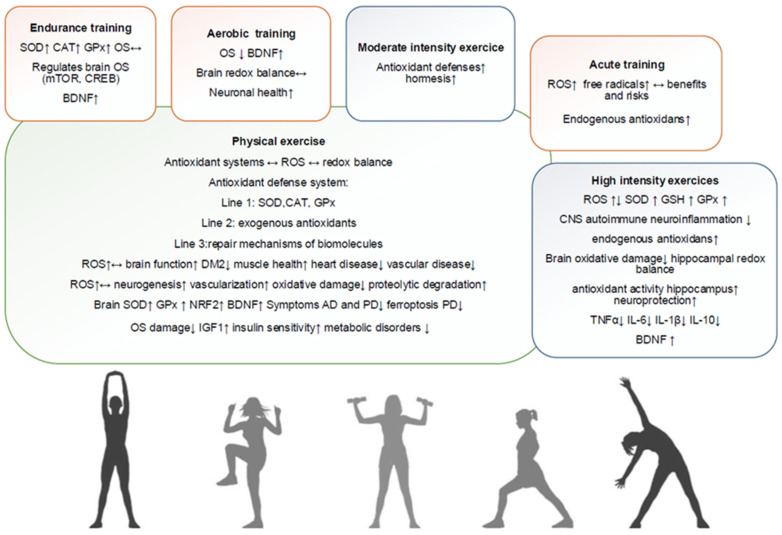
Sport activities and oxidative stress in brain function.

Lee et al. suggest that ROS may be essential for cell function, although their precise role remains poorly understood. Their study shows that antioxidant systems may facilitate the controlled release of ROS, which is necessary for normal brain function. ROS, although commonly associated with oxidative damage, appears to play a beneficial role in physiological processes (Figure 3), contributing to cell signaling and maintaining redox balance [118].

To counteract the damaging effects of ROS, the body has developed a complex antioxidant defense system structured along three lines. The first line of defense, consisting of antioxidant enzymes such as superoxide dismutase (SOD), catalase (CAT) and glutathione peroxidase (GPx), plays a key role in neutralizing free radicals, thereby protecting cells from oxidative damage. The second line of defense relies on exogenous antioxidants from the diet, which complement the action of enzymes and help maintain oxidative balance. The third line of defense involves the repair mechanisms of damaged biomolecules, thus ensuring cellular integrity and the body’s functionality in the face of OS [119].

Radak et al. claim that exercise increases the production of ROS but paradoxically improves muscle health and reduces the risk of heart disease, vascular disease, certain cancers and type II diabetes [2,120]. Regular exercise also has beneficial effects on brain function, with preventive and therapeutic roles in stroke, AD and PD. These effects include neurogenesis, increased vascularization, reduced oxidative damage and enhanced proteolytic degradation [121,122,123].

Kawamura and Muraoka highlighted the dual role of ROS and free radicals generated during acute exercise, with both health benefits and risks. While it is widely accepted that exercise elevates free radical levels, directly measuring these species is challenging due to their reactivity and short half-life; thus, researchers often rely on indirect markers of OS. The degree of exercise-induced oxidative responses can vary significantly among individuals, complicating the interpretation of findings and necessitating a thorough examination of individual redox status. Moreover, the authors emphasize that the sources of ROS extend beyond skeletal muscle to include blood and other organs, suggesting that a comprehensive, integrative approach is essential for a complete understanding of the underlying mechanisms [124].

Meng and Su (2024) conducted a review on how exercise affects oxidative and nitrosative stress, focusing on antioxidants. By analyzing data from 41 studies published between 2004 and 2024, they found that moderate exercise enhances the body’s antioxidant defenses through hormesis, while excessive exercise increases OS. Although natural dietary antioxidants are beneficial, high-dose supplements may counteract positive adaptations to exercise. The review emphasizes the need for personalized exercise and nutrition plans, considering factors like age, gender, ethnicity and socioeconomic status to optimize health outcomes for athletes and the general population [125].

Powers et al. (2023) also conducted a systematic review, one that examined the impact of endurance training on OS and antioxidant enzyme activity in skeletal muscle. Their findings reveal that endurance training significantly boosts the activity of SOD, CAT and GPx, which are crucial for protecting against exercise-induced oxidative negative activity. Preclinical studies indicate that resistance training enhances the abundance and activity of SOD, including SOD1 and GPx in rodent skeletal muscle. Clinical evidence further supports that resistance training increases the activity of SOD1, SOD2, GPx and CAT in human muscle, underscoring its role in improving muscle antioxidant capacity. While endurance and high-intensity interval training (HIIT) also increase these enzyme activities, studies in this domain are limited, indicating a pressing need for further research [126].

In early studies, Rybak, Somani et al. provided valuable insights into the effects of exercise training on the antioxidant systems within various brain regions of rats. Their findings reveal significant differences in the levels of glutathione (GSH), glutathione disulfide (GSSG) and the activities of antioxidant enzymes, including SOD and GPx. Notably, regions such as the brainstem and corpus striatum demonstrated marked increases in SOD and GPx activity following exercise training [127]. More recently, Zaychik et al. (2021) investigated the neuroprotective effects of high-intensity continuous training (HICT) against autoimmune neuroinflammation, particularly in the context of MS and its experimental autoimmune encephalomyelitis model (EAE). Healthy mice performed HICT on a treadmill before being injected with encephalitogenic T cells to induce EAE. Mice undergoing HICT exhibited milder EAE symptoms compared to sedentary mice, with reduced clinical severity and less infiltration of T cells and neurotoxic macrophages/microglia, as well as reduced myelin and axonal loss. HICT reduced the number of resident microglia without changing their profile and led to decreased ROS production and decreased secretion of proinflammatory cytokines such as IL-6 and monocyte chemoattractant protein. HICT protects the CNS from autoimmune neuroinflammation by decreasing microglial-derived ROS and neurotoxic responses, emphasizing the role of exercise intensity in neuroprotection and its potential therapeutic implications for MS [128].

Although several studies over the past decade have begun to explore the neuroprotective effects of resistance training, few have specifically addressed its relationship with OS. This indicates a need for further investigation to fully understand how resistance exercise influences oxidative activity and brain health.

Lu et al. (2021) conducted a systematic review that examined the effects of high-intensity exercise (HIE) on OS and antioxidant status in untrained individuals. After analyzing 21 studies, they found strong evidence that acute oxidative reactions occur immediately after HIE, but this effect is transient and usually recovers within 24 h due to the activation of endogenous antioxidant systems. Higher fitness levels correlate with lower OS, indicating that regular physical activity improves antioxidant capacity and overall health. The intensity and duration of HIE are critical factors influencing OS levels, while individual characteristics such as fitness level and age also play significant roles. HIE can promote health in untrained individuals, with recommended intensities above 70% VO₂max recommended for effective antioxidant enhancement [129].

Aerobic physical training has been extensively studied in both humans and animals, demonstrating its effectiveness in reducing OS, maintaining brain redox balance and increasing levels of BDNF [130,131,132]. The impact of aerobic exercise on the brain’s redox system is well-documented, highlighting its beneficial effects on oxidative responses and neuronal health. However, the cerebral effects of resistance exercise remain less clear, as research in this area has only gained traction in recent years [133,134,135,136].

Freitas et al. (2018) investigated the effects of six weeks of HIIT on OS, inflammatory markers and neurotrophic factors in the hippocampus of young male Wistar rats. The results showed that HIIT reduced oxidative damage and increased both enzymatic (superoxide dismutase) and non-enzymatic antioxidant defenses in the hippocampus. Additionally, HIIT decreased pro-inflammatory cytokines (TNFα, IL-6, IL-1β and IL-10) while enhancing BDNF levels. The findings suggest that HIIT positively affects hippocampal redox balance by reducing oxidative stress and inflammation and increasing antioxidant activity and neurotrophic support. This is the first study to demonstrate the benefits of HIIT in reducing hippocampal OS and promoting neuroprotection [137].

Rocha-Gomes et al. (2023) explored the effects of HII on behavior and hippocampal neurochemistry in ovariectomized adult rats, a model for menopause-related changes. The rats were divided into four groups: sedentary with or without ovariectomy (SHAM-SED and OVX-SED) and trained with or without ovariectomy (SHAM-HIIT and OVX-HIIT). The results indicated that the OVX-SED group exhibited low levels of BDNF and antioxidant enzymes, correlating with memory impairments. In contrast, the HIIT groups showed increased BDNF levels and antioxidant activity in the hippocampus, which improved memory performance. However, HIIT also resulted in elevated plasma corticosterone levels and increased anxiety-like behaviors, suggesting that while HIIT can enhance cognitive function in ovariectomized rats, it may also provoke stress-related responses [138].

While the specific cellular mechanisms by which resistance exercise regulates brain OS are not yet fully understood, there are reasons to believe that the adaptive changes induced by resistance training in muscle may lead to the up-regulation of antioxidant defenses and the regulation of brain redox balance through various proteins and pathways [125,139]. Key players in this process include the mammalian target of rapamycin (mTOR), a serine/threonine kinase essential for cell growth, proliferation and survival in the brain, and the CAMP-response element-binding protein (CREB), which regulates the expression of genes critical for dopaminergic neuron function [140,141]. Both mTOR and CREB facilitate enhanced translation initiation through the phosphorylation of protein kinase B (AKT), resulting in increased expression and activation of BDNF in both muscle and brain. These pathways suggest a complex interplay between resistance training, OS regulation and neurotrophic support in promoting brain health [142,143,144].

Li et al. (2022) demonstrate that BDNF plays crucial roles in the CNS by activating two main receptors: the tropomyosin receptor kinase B (TrkB) and the p75 neurotrophin receptor (p75NTR). It is involved in a variety of physiological processes, including neurogenesis, synaptic plasticity and neuronal survival. Altered BDNF levels have been implicated in the progression of diseases, highlighting its potential role in both disease mechanisms and therapeutic strategies [145].

These pathways collectively enhance BDNF secretion and contribute to the beneficial effects of resistance exercise on brain function by activating nuclear factor Nrf2, which regulates the expression of various detoxification enzymes and antioxidants. This regulation is essential for protecting brain cells from oxidative damage, maintaining mitochondrial function and ensuring cellular redox balance. In response to OS or electrophilic modifications, Nrf2 translocates to the nucleus and binds to antioxidant response elements (ARE) in DNA, promoting the transcription of cytoprotective genes like heme oxygenase-1 (HO-1), SOD and glutathione S-transferase (GST). This mechanism highlights the interplay between protective cellular pathways, underscoring the potential of physical activity in enhancing brain health and resilience against oxidative stress [146,147].

The release of BDNF during muscle contraction plays a critical role in neuronal signaling and neuroprotection. When BDNF reaches the brain, it binds to the TrkB, initiating a cascade of signaling pathways, including PI3K/AKT/mTOR, PI3K/AKT/CREB, PI3K/extracellular signal-regulated kinase (ERK)/CREB, and phospholipase Cγ (PLCγ)/Calcium/Calmodulin-dependent protein kinase II (CamKII)/CREB [148,149,150,151].

Recent studies have shown that the PI3K/AKT signaling pathway plays a crucial role in both neurodegenerative diseases and mood disorders, influencing processes such as cell survival, neuroinflammation and synaptic plasticity. In depression, its activation may help restore neurotransmitter balance and promote neurogenesis, suggesting potential as an antidepressant target. For neurodegenerative diseases such as PD and AD, the pathway provides neuroprotection by reducing oxidative stress, apoptosis and inflammation, thereby maintaining neuronal health. Natural compounds that activate PI3K/AKT show promise for treating these diseases, particularly in protecting dopamine-producing neurons in Parkinson’s disease and counteracting pathological signs in AD [152,153,154].

Physical exercise stimulates the secretion of growth factors like IGF1, which activate the PI3K/AKT pathway, promoting cell growth, survival and metabolism. In skeletal muscle, activation of PI3K/AKT enhances glucose uptake through the translocation of insulin-regulated glucose transporter (GLUT4); improving insulin sensitivity is crucial for managing conditions like insulin resistance and type 2 diabetes. PI3K/AKT activation during exercise supports muscle growth, increases insulin sensitivity, promotes cardiovascular health and mitigates metabolic disorders [155,156,157,158].

Souza et al. (2022) highlighted the crucial role of the Nrf2 and the ARE signaling pathway in regulating cellular antioxidant defenses, which are essential for maintaining mitochondrial function and homeostasis. Physical exercise enhances this pathway, particularly in the brain and skeletal muscle, though the underlying mechanisms are not fully understood. Increasing evidence suggests that the tetrahydrobiopterin (BH4) pathway and epigenetic changes, such as DNA methylation, contribute to the exercise-induced antioxidant response. Activation of Nrf2 by exercise has been linked to protective cellular responses, including antioxidant production, anti-inflammatory effects and mitochondrial enhancement, making it a promising therapeutic avenue for neurodegenerative diseases. Regular physical exercises have been shown to improve symptoms of conditions like PD and AD, supporting its role as a non-pharmacological tool for neuroprotection [159].

Thirupathi et al. (2024) showed that PD involves OS, contributing to the loss of dopaminergic neurons in the brain, with ferroptosis (an iron-dependent cell death process) playing a role due to imbalances in the brain’s redox state. Exercise helps reduce oxidative damage by activating Nrf2 and BDNF signaling pathways, which regulate antioxidant defense. This antioxidant effect of exercise may attenuate ferroptosis, potentially slowing disease progression [160].

## 8. Oxidative Stress and Vagal Stimulation

The relationship between OS and vagal stimulation is approached nowadays in the context of its therapeutic potential. Vagal stimulation has been shown to elicit protective effects against oxidative reactions in various organ systems, for example, in studies with rat models of myocardial ischemia, with significantly reduced ROS production, implicating the involvement of the NADPH oxidase (Nox) pathway as a protective effect [161]. In this case, the authors suggest that vagal stimulation activates the AMP-activated protein kinase (AMPK) and protein kinase C (PKC) pathways, which are crucial for cellular energy homeostasis and redox balance. This finding aligns with the notion that vagal nerve activity can modulate oxidative stress responses, potentially offering a therapeutic avenue for conditions characterized by oxidative damage.

Vagal stimulation has also been shown to influence OS in the context of inflammation, as cholinergic stimulation via vagal pathways improved oxidative responses and inflammation in experimental myocardial infarction models [162]. The study highlighted that vagal stimulation led to increased activity of antioxidant enzymes such as SOD and GPx, which play critical roles in decreasing oxidative damage. This suggests that vagal stimulation may enhance the antioxidant defense mechanisms in tissues subjected to oxidative stress.

It is well known that the autonomic nervous system, particularly the parasympathetic branch mediated by the vagus nerve, plays a crucial role in regulating physiological responses to stress. The vagal nerve stimulation can modulate autonomic responses, thereby influencing OS levels in the body [163].

Interventions such as transcutaneous vagal nerve stimulation (tVNS) have demonstrated significant effects on emotional regulation and stress responses, which are closely linked to oxidative stress pathways. Moreover, non-invasive vagal nerve stimulation reduces sympathetic reactivity in patients with post-traumatic stress disorder (PTSD), suggesting a potential mechanism by which vagal stimulation can mitigate stress-induced oxidative damage [164]. By enhancing vagal tone, these interventions may help restore balance in the autonomic nervous system, thereby reducing the overall oxidative burden on the body, hence with clinical implications, such as modulating gut injury or lung permeability in trauma-hemorrhagic shock models, which are often associated with elevated OS [165]. The protective effects observed are linked to a reduction in the inflammatory cytokine response, further supporting the role of vagal stimulation in reducing oxidative activity. Even subdiaphragmatic vagal nerve stimulation seems to attenuate the development of hypertension, a condition often exacerbated by OS, with implications for the risk of hemorrhagic stroke [166]. The modulation of the nucleus of the solitary tract transcriptional networks suggests that vagal stimulation may influence central nervous system pathways that regulate cardiovascular responses and OS levels.

Heart failure, diabetes and other neurodegenerative diseases are currently being considered for addressability in terms of modulating chronic OS. The development of non-invasive vagal stimulation techniques, such as tVNS, presents an exciting opportunity for clinical applications since non-invasive methods can access vagal nerve projections and may provide a feasible approach to modulating oxidative responsivity in various patient populations [167].

The cholinergic anti-inflammatory pathway has also been proposed as a mechanism through which vagal stimulation can influence OS and inflammation, providing a functional connection between the immune and central nervous systems and allowing the modulation of inflammatory responses that are often exacerbated by oxidative damage [168]. This suggests that by reducing systemic inflammation, vagal stimulation may indirectly alleviate oxidative influence in neurological disorders.

The antioxidant properties of various medicinal plants, including Withania somnifera, may complement vagal stimulation in treating oxidative stress-induced neurobehavioral disorders [169]. The combination of herbal interventions with vagal stimulation could enhance antioxidant defenses and improve neurological outcomes. Furthermore, vagus nerve stimulation can enhance neuroplasticity and cognitive function in animal models of traumatic brain injury [170]. Hence, vagal stimulation may promote recovery and adaptation in the nervous system.

The relationship between OS and vagal stimulation has been highly studied in drug-resistant cases of epilepsy, especially throughout the last two decades. ROS production can lead to exacerbating seizure activity and contribute to the development of epilepsy-related comorbidities. by promoting excitotoxicity, which is a critical mechanism underlying seizure onset [171]. High levels of oxidative stress markers, such as malondialdehyde (MDA) and decreased antioxidant enzyme activity, indicate an imbalance that may also facilitate seizure activity [171,172].

The role of OS in specific types of epilepsy, such as Dravet syndrome and other epileptic encephalopathies, has been documented by assessing the severity of seizures and cognitive impairment in these conditions, suggesting that targeting OS could be a viable therapeutic strategy [173]. Furthermore, the relationship between oxidative impact and inflammation in epilepsy is significant, as inflammatory processes can further exacerbate oxidative damage, creating a vicious cycle that complicates seizure management [174].

Vagal nerve stimulation (VNS) has emerged as a promising adjunctive therapy for patients with drug-resistant epilepsy. Approved by the FDA in 1997, VNS has been shown to reduce seizure frequency in a substantial proportion of patients, with studies indicating a greater than 50% reduction in seizures for about 55% of individuals [175,176].

The mechanisms by which VNS exerts its effects are multifaceted. Vagal stimulation is believed to modulate neurotransmitter release, enhance neuroplasticity and reduce excitability in seizure-prone brain regions. VNS may also desynchronize abnormal electrical activity in the brain, thereby decreasing seizure frequency and severity [177]. This modulation of brain activity may also influence OS levels, as vagal stimulation has been associated with improved antioxidant defenses and reduced inflammation [178].

The interaction between OS and vagal stimulation in epilepsy is an emerging area of research on the potential neuroprotective effects. VNS can lead to a reduction in OS markers in patients with drug-resistant epilepsy [179].

The potential for VNS to influence the autonomic nervous system may also play a role in managing oxidative responsivity. By enhancing vagal tone, VNS may help restore balance in the autonomic nervous system, which is often dysregulated in individuals with epilepsy. This restoration could lead to improved OS responses and overall brain health [180,181].

An interesting perspective is brought by Kishi and Hirooka about the activation of the sympathetic nervous system (SNS), a crucial event in hypertension, along with OS primarily produced by angiotensin II type 1 (AT(1)) receptor and nicotinamide adenine dinucleotide phosphate (NAD (P) H) oxidase in the brain. The authors investigated the role of OS in the rostral ventrolateral medulla (RVLM) in hypertensive rats, revealing that AT(1) receptor and NAD (P) H oxidase-induced OS cause sympathoexcitation. Environmental factors like high salt intake and calorie diet may increase OS, increasing hypertension risk [182].

Another review by Chan YHJ and Chan HHS also deals with the RVLM, which, affected by oxidative and nitrosative stress, leads to neurogenic hypertension and expands our understanding of brain stem death. The authors suggest that these stressors are not interchangeable and that future antioxidant therapies should consider sympathetic vasomotor tone generation, maintenance, regulation or modulation. This shift towards nitrosative stress in the RVLM may provide new therapeutic interventions to slow down the progression toward brain stem death [183].

## 9. Final Considerations on Oxidative Stress and Dietary Factors

The global rise in neurological diseases has been associated with dietary changes, particularly in industrialized nations, contributing to an increase in PD, AD, MS and other neurodegenerative conditions. As shown up to this point, dietary choices may influence neurotransmitter function, hence controlling brain energy metabolism and diminishing OS.

Diet greatly affects memory and cognitive function, as certain foods and supplements, such as omega-3 polyunsaturated fatty acids, walnuts, berries and flavonoids, can markedly improve mental performance and memory. Polyphenols participate in brain metabolism via anti-inflammatory pathways [184]. The processes that convert dietary energy into neural activity are essential for optimal brain function. Energy regulation in neurons can affect synaptic activity, hence altering metabolic energy. It denotes a bidirectional link wherein cognitive processes affect physiological functions at a cellular level [185,186]. Nutrients enable various critical functions of the brain, such as sustaining neuronal health and viability, generating nerve impulses, synthesizing neurotransmitters, maintaining lipid membrane asymmetry and integrity, supporting synaptic plasticity, and regulating metabolic processes related to energy production. These mechanisms are essential for cerebral health and are intricately associated with cognitive function [186].

Certain foods are correlated with diminished cognitive function, including saturated fats, which are associated with the later development of dementia, and high-fat dairy products, which have been linked to reduced cognitive performance and an elevated risk of cognitive decline [187]. On the other hand, proteins and amino acids derived from lean meat and fish serve as precursors for essential neurotransmitters, including serotonin, dopamine and norepinephrine, which are vital to the regulation of mood, motivation and attention. Also, acetylcholine and glutamate, prevalent in seafood, are essential for learning and memory functions [186].

Whole grains and legumes provide complex carbs, fiber and vitamin E and are overall linked to a reduced risk of dementia; nevertheless, the intake of refined cereals and grains correlates with diminished cognitive performance and increased cognitive decline [186].

B vitamins are also essential for cognitive function, as they facilitate inhibition and diminish excessive excitement, lowering anxiety and depression rates. Niacin (vitamin B3) comprises foods containing tryptophan: meat, whole grains and dairy products, peanuts, salmon and mushrooms. Consistent coffee usage also enhances niacin intake. Optimal niacin levels are essential, as a lack may result in pellagra, characterized by dementia, dermatitis, diarrhea and sometimes death. Niacin was even utilized for dyslipidemia management before the widespread adoption of statins in doses of up to 3000 mg/day [188,189]. Specific measures should be considered for a healthy diet when it comes to tryptophan, as its absorption happens in the small intestine, and concurrent consumption of other significant neutral amino acids may restrict its absorption and availability. Dietary carbohydrates can diminish tryptophan levels by stimulating insulin secretion, which facilitates the preferential uptake of non-tryptophan amino acids. Mostly bound to albumin, tryptophan traverses the BBB and penetrates the CNS via the L-type amino acid transporter, which aids in the transport of large neutral amino acids [190].

Vitamin B6 (pyridoxal, pyridoxamine and pyridoxine) is crucial for the regulation of cognitive function and emotional state. Its shortage correlates with increased blood homocysteine levels, a risk factor for cerebrovascular illness. Deficiency in Vitamin B6 has also been associated with neuropsychiatric disorders, including seizures, migraines, chronic pain or depression. Deficient levels of vitamin B6 are prevalent in the elderly population, with the risk that associated hyperhomocysteinemia would be implicated in the etiology of dementia, requiring supplementation [191].

Although vitamin B6 deficiency is rare, subclinical deficiency is still common, potentially resulting in diminished cognitive function, as it serves as an antioxidant by directly neutralizing free radicals and indirectly bolstering the glutathione antioxidant defense system. A shortage in vitamin B6 may impair neurotransmitter metabolism, particularly gamma-aminobutyric acid and serotonin, within the CNS [191].

The buildup of ceramides and cholesterol esters due to OS has been associated with motor neuron degeneration, suggesting that lipid metabolism contributes to the disease mechanism alongside genetic factors that affect the CNS [192]. Therefore, mitochondrial malfunction and the formation of ROS, toxic to motor neurons in amyotrophic lateral sclerosis (ALS), are strongly associated with mutations in key genes, including SOD1 [193]. Mutations in SOD1 are associated with about 20 percent of familial ALS [194]. Besides SOD1, mutations in the FUS and TDP-43 genes can impair RNA metabolism and protein homeostasis, hence intensifying oxidative damage in motor neurons [195,196,197]. The interaction between these genetic changes and OS establishes a negative pattern in which oxidative damage causes neuronal impairment, further exacerbating oxidative responses due to compromised cellular repair mechanisms [198,199]. Dietary strategies aimed at reducing OS in ALS include the inclusion of polyphenol-rich foods, adherence to anti-inflammatory dietary regimens like the Mediterranean diet, and the possible application of NAD^+^ supplementation [200,201]. These techniques seek to improve antioxidant defenses and reduce oxidative damage, which is essential in the management of ALS.

Along with the dietary aspects for the brain, discussed over the PD, AD, MS and cerebrovascular disease topics, the interplay between OS and dietary components will remain complex, with data indicating that dietary antioxidants play a protective role in enhancing brain health. In terms of preventive medicine, a diet abundant in fruits, vegetables and other sources of antioxidants might alleviate OS and inflammation, potentially diminishing the risk of neurodegenerative disorders. Mechanisms via which food patterns affect oxidative reactions also start pinpointing specific dietary interventions designed for neuroprotection.

## 10. Conclusions

We consider that the timing and quantity of antioxidant administration are crucial elements that can affect treatment outcomes, highlighting the necessity for well-structured clinical trials to determine effective procedures.

The interplay between OS, mitochondrial dysfunction and neuroinflammation underscores the complexity of PD and other central disorders pathogenesis, as well as future implications that extend beyond neurodegeneration. Neuroprotection is assessable not only by means of innovative pharmacological developments but also by the use of natural compounds with antioxidant properties, which have shown promise in reducing oxidative aggression and improving outcomes within the concept of preventive medicine.

Therapeutic strategies, doubled by dietary and physical changes in lifestyle, targeting oxidative stress will continue to be explored for their neuroprotective effects in relation to neurodegenerative patterns.

## Abbreviations

The following abbreviations are used in this manuscript:

OS	Oxidative stress
ROS	Reactive oxygen species
PD	Parkinson’s disease
AD	Alzheimer’s disease
MS	Multiple sclerosis
CNS	Central nervous system
NMDAR	N-methyl-d-Aspartate receptor
RNS	Reactive nitrogen species
Nrf2	Nuclear factor erythroid 2-related factor 2
PON2	Paraoxonase 2
PI3K/Akt	Phosphoinositide 3-kinase
BDNF	Brain-derived neurotrophic factor
Epo	Erythropoietin
Gpi	Globus pallidus internus
SNr	Substantia nigra pars reticulata
Gpe	Globus pallidus externus
SIRT1	Sirtuin 1
NAC	N-acetylcysteine
VDR	Vitamin D receptor
MAPKs	Mitogen-activated protein kinases
NADPH	Nicotinamide adenine dinucleotide phosphate
MDA	Malondialdehyde
BBB	Blood–brain barrier
ICH	Intracerebral hemorrhage
HO-1	Oxygenase-1
LTP	Long-term potentiation
SOD	Superoxide dismutase
CAT	Catalase
GPx	Glutathione peroxidase
HIIT	High-intensity interval training
GSH	Glutathione
GSSG	Glutathione disulfide
HICT	High-intensity continuous training
EAE	Autoimmune encephalomyelitis
HIE	High-intensity exercise
mTOR	Mammalian target of rapamycin
CREB	CAMP-response element-binding protein
TrkB	Tropomyosin receptor kinase B
ARE	Antioxidant response elements
GST	Glutathione S-transferase
GLUT4	Insulin-regulated glucose transporter
BH4	Tetrahydrobiopterin
Nox	NADPH oxidase
AMPK	AMP-activated protein kinase
PKC	Protein kinase C
tVNS	Transcutaneous vagal nerve stimulation
PTSD	Post-traumatic stress disorder
VNS	Vagal nerve stimulation
SNS	Sympathetic nervous system
AT(1)	Angiotensin II type 1
NAD (P) H	Nicotinamide adenine dinucleotide phosphate
RVLM	Rostral ventrolateral medulla
ALS	Amyotrophic lateral sclerosis
